# Non-immunological complications following kidney transplantation

**DOI:** 10.12688/f1000research.16627.1

**Published:** 2019-02-18

**Authors:** Abraham Cohen-Bucay, Craig E. Gordon, Jean M. Francis

**Affiliations:** 1 Department of Nephrology and Mineral Metabolism, Instituto Nacional de Ciencias Médicas y Nutrición Salvador Zubirán, Mexico City, 14080, Mexico; 2 Nephrology Department, American British Cowdray Medical Center, Mexico City, 05300, Mexico; 3 Division of Nephrology, Tufts Medical Center, Boston, MA, 02111, USA; 4 Renal Section, Boston University Medical Center, Boston, MA, 02118, USA

**Keywords:** kidney transplant, post-transplant complications

## Abstract

Kidney transplantation (KT) is the most effective way to decrease the high morbidity and mortality of patients with end-stage renal disease. However, KT does not completely reverse the damage done by years of decreased kidney function and dialysis. Furthermore, new offending agents (in particular, immunosuppression) added in the post-transplant period increase the risk of complications. Cardiovascular (CV) disease, the leading cause of death in KT recipients, warrants pre-transplant screening based on risk factors. Nevertheless, the screening methods currently used have many shortcomings and a perfect screening modality does not exist. Risk factor modification in the pre- and post-transplant periods is of paramount importance to decrease the rate of CV complications post-transplant, either by lifestyle modification (for example, diet, exercise, and smoking cessation) or by pharmacological means (for example, statins, anti-hyperglycemics, and so on). Post-transplantation diabetes mellitus (PTDM) is a major contributor to mortality in this patient population. Although tacrolimus is a major contributor to PTDM development, changes in immunosuppression are limited by the higher risk of rejection with other agents. Immunosuppression has also been implicated in higher risk of malignancy; therefore, proper cancer screening is needed. Cancer immunotherapy is drastically changing the way certain types of cancer are treated in the general population; however, its use post-transplant is limited by the risk of allograft rejection. As expected, higher risk of infections is also encountered in transplant recipients. When caring for KT recipients, special attention is needed in screening methods, preventive measures, and treatment of infection with BK virus and cytomegalovirus. Hepatitis C virus infection is common in transplant candidates and in the deceased donor pool; however, newly developed direct-acting antivirals have been proven safe and effective in the pre- and post-transplant periods. The most important and recent developments on complications following KT are reviewed in this article.

## Introduction

End-stage renal disease (ESRD) is one of the leading causes of premature mortality, and the death rate for patients on dialysis is 166 per 1,000 patient-years ^1^. Kidney transplant is the best treatment of ESRD and decreases the mortality rate to 29 per 1,000 patient-years ^1^. Despite this, kidney transplant recipients still experience a high incidence of complications in the post-transplant period. On one hand, the pre-transplant period, in which the patients had a very low glomerular filtration rate (GFR) and were on renal replacement therapy, inherently confers a high risk of complications which are not completely reversed by a kidney transplant. On the other hand, new factors are added in the post-transplant period, most notably immunosuppressive medications and their side effects. Therefore, the post-transplant period is associated with a wide range of complications, including cardiovascular (CV), metabolic, oncologic, infectious, immunological, surgical, osseous, and hematologic complications. This review will focus on CV, metabolic, oncologic, and infectious complications with an emphasis on areas with important developments in recent years. We chose to review these particular complications because of their frequency and associated mortality in kidney transplant recipients ( Table 1). Much of the management and screening of post-kidney transplant complications occurs while the patients are being evaluated for kidney transplant candidacy in the pre-transplant period, which will be reviewed here alongside specific post-transplant management.

**Table 1.** Incidence and mortality associated with cardiovascular, metabolic, oncologic, and infectious complications in kidney transplant recipients.

Cardiovascular	• ~30% of overall mortality in kidney transplant patients ^16, 17^ • Cumulative incidence of MI at 3 years: 4.2%–11.1% ^6, 7^ • Mortality: CAD 4.1 (3.0–5.6) /100PY, no CAD 1.9 (1.6–2.1)/100PY ^17^
Diabetes mellitus	• Incidence of PTDM: about 12% in 5 years ^16^ • Mortality: DM1 2.3 (1.3–3.7)/100PY, DM2 3.7 (2.7–5.0)/100PY, no DM 1.8 (1.6–2.1)/100PY ^17^ • Increases mortality due to CV (RR 1.5) and infectious (RR 1.87) causes ^18^
Cancer	• 24% of overall mortality in kidney transplant patients ^17^ • Transplantation increases twofold the risk of cancer ^19^
Infections	• 13% of overall mortality in kidney transplant patients ^17^

/100PY, per 100 patient-years; CAD, coronary artery disease; CV, cardiovascular; DM1, diabetes mellitus type 1; DM2, diabetes mellitus type 2; MI, myocardial infarction; PTDM, post-transplantation diabetes mellitus; RR, relative risk.

## Cardiovascular disease

Chronic kidney disease (CKD) is an independent risk factor for atherosclerotic coronary artery disease (CAD), and CAD incidence and severity increase as the GFR declines ^2, 3^. By the time patients reach ESRD, the prevalence of coronary artery stenosis ranges from 37% to 58% in asymptomatic patients ^4^.

Although kidney transplant is the most effective way to decrease the risk of CV events, CV death still accounts for 30% of overall mortality, the most common cause of death in the post-transplant period ^5^. The cumulative incidence of myocardial infarction in the post-transplant period is 4.2% to 11.1% at 3 years ^6, 7^. Therefore, two strategies are commonly used to decrease the incidence of CV disease in post-transplant patients: (1) pre-transplant screening with subsequent treatment and exclusion of very high-risk patients from listing and (2) risk factor modifications in the pre- and post-transplant period.

The most important CV risk factors in the ESRD population include diabetes mellitus (DM), prior CV disease (including stroke and peripheral arterial disease), dialysis vintage of more than 1 year, left ventricular hypertrophy, age greater than 60 years, smoking, hypertension, and dyslipidemia ^5^. Many of these risk factors persist in the post-transplant period but others can develop *de novo*, including post-transplantation diabetes mellitus (PTDM) (previously known as new-onset diabetes after transplantation), drug-induced hypertension, drug-induced dyslipidemia, proteinuria, and chronic inflammation ^8, 9^. Non-traditional CV risk factors in the post-transplant period include intrarenal resistive index (RI) greater than 0.80, which is associated with higher risk of death ^10^. Interestingly, the RI during protocol biopsies correlates more with recipients’ factors (for example, age and central hemodynamic factors) than with graft or histologic factors ^10^. Hypotension in the pre-transplant period might also be a risk factor for adverse outcomes in the post-transplant period, evidenced by recent studies associating the use of midodrine in the pre-transplant period with graft failure, death, and major adverse cardiovascular events (MACE) post-transplant ^11, 12^.

Identification and optimization of modifiable risk factors are of utmost importance for prevention of CV events post-transplant. Lifestyle modifications, including exercise, smoking cessation, and maintenance of a healthy weight, are always recommended. Treatment of all post-transplant patients with a statin is suggested by the Kidney Disease Improving Global Outcomes (KDIGO) guidelines on lipid management (grade 2A) ^13^ based on the ALERT (Assessment of Lescol in Renal Transplantation) trial, which showed that fluvastatin decreases the risk of MACE in kidney transplant patients ^14, 15^. Treatment with renin–angiotensin–aldosterone system blockade to decrease proteinuria and hence to decrease CV risk has not proven to be as effective as in the general population ^20– 22^, but larger prospective studies are needed before reaching a definitive conclusion.

The ideal screening strategy is unknown and differs across transplant centers. Current clinical guidelines are based mostly on expert opinion with a low level of evidence, but the general recommendation is the use of a risk-stratified approach in which non-invasive techniques are used first, and coronary angiography is reserved for high-risk patients or when the non-invasive tests are abnormal ^5, 23– 26^. However, clinicians need to be aware that the pre-transplant CV evaluation should not be the same as for any other non-cardiac surgery, particularly because of the high risk of severe allograft dysfunction (in 6% to 33% of patients) and allograft loss (in 3% to 12% of patients) if cardiac surgery is required in the post-transplant period ^27– 30^. Therefore, it is very important to note that the goal of the screening strategy is to decrease the CV events and mortality not only in the perioperative period but also in the long term ^5^.

The risk-stratified approach has proven beneficial for low-risk patients in whom the event rate is very low and invasive tests are therefore unnecessary ^4^. This strategy is more controversial for intermediate- and high-risk patients for several reasons. First, the accuracy of stress tests compared with that of coronary angiography is limited ( Table 2). In a 2011 meta-analysis, the pooled sensitivity and specificity for dobutamine stress echocardiography (DSE) were 0.79 (95% confidence interval (CI) 0.67–0.88) and 0.89 (95% CI 0.81–0.94), respectively. For myocardial perfusion studies (MPS), the pooled sensitivity was 0.74 (95% CI 0.54–0.87) and pooled specificity was 0.70 (95% CI 0.51–0.84) ^31^. Second, the prognostic value of non-invasive tests has been the subject of controversy. In a 2015 meta-analysis, Wang *et al*. ^32^ reported that patients with an abnormal DSE have a higher risk of all-cause mortality and MACE but that patients with an abnormal MPS have a higher risk of MACE. However, both tests predicted outcomes poorly: a substantial number of patients with an abnormal non-invasive test did not develop adverse outcomes, while a large proportion of patients who had a normal non-invasive test had adverse cardiac outcomes.

**Table 2.** Accuracy of non-invasive cardiovascular tests compared with coronary angiography in kidney transplant candidates.

Test	Sensitivity	Specificity	Notes
DSE ^31^	0.79 (0.67–0.88)	0.89 (0.81–0.94)	Meta-analysis of 13 studies (n = 745)
MPS ^31^	0.74 (0.54–0.87)	0.70 (0.51–0.84)	Meta-analysis of nine studies (n = 582)
CACS ^33– 36^	0.54–0.92	0.44–0.87	Four studies, n ranging from 18 to 148. Total n = 309. Different cutoff values were used in each study.
Coronary CT ^34, 39^	0.65–0.80	0.74–0.86	Two studies, n ranging from 19 to 147. Total n = 147. The larger study reported sensitivity/specificity of combining three CV risk factors plus coronary CT.

CACS, coronary artery calcium score; CT, computed tomography; CV, cardiovascular; DSE, dobutamine stress echocardiogram; MPS, myocardial perfusion scan.

The same meta-analysis ^32^ showed that an abnormal coronary angiography was associated with increased risk for all-cause mortality (odds ratio [OR] 2.96, 95% CI 1.25–7.00) and MACE (OR 16.02, 95% CI 2.42–105.98). However, coronary angiography was not superior to non-invasive testing at predicting future adverse CV events. Nevertheless, the predictive ability of coronary angiography might have been falsely reduced by revascularization, which significantly decreases MACE (OR 0.19, 95% CI 0.05–0.72) and all-cause mortality (OR 0.28, 95% CI 0.12–0.64) ^32^. These results might have also been affected by referral bias given that many of the patients who underwent coronary angiography had an abnormal non-invasive test previously. When revascularization is indicated, this should occur prior to transplantation ^5^. However, clinicians need to take into account the need for antiplatelet or anticoagulation treatment after revascularization, which could delay or postpone transplantation.

Therefore, a perfect screening test for CV disease in kidney transplant candidates does not exist and all of the existing strategies have deficiencies. Newer CV tests, including the coronary artery calcium score (CACS) and coronary computed tomography (CT), are now being studied in the ESRD population. The correlation between CACS and angiographic CAD in the CKD/ESRD population is uncertain ^33– 36^ given that high calcium scores may reflect medial instead of intimal vascular calcifications ( Table 2). Nevertheless, CACS has been studied prospectively in two trials in kidney transplant candidates with conflicting results regarding the ability of CACS to predict MACE and mortality in comparison with MPS ^37, 38^. Therefore, at this time, the CACS cannot be recommended as a first-line strategy for CV disease screening pre-kidney transplant.

Coronary CT has good correlation with angiographic coronary disease ^34, 39^, and an abnormal coronary CT is an independent risk factor for adverse CV outcomes in kidney transplant candidates ^38, 40^. These results are from relatively small studies and require validation in larger trials with a more diverse population before the widespread use of coronary CT can be recommended as a CV disease screening method in renal transplant candidates. Furthermore, there is a safety concern of performing coronary CT in patients with residual kidney function given the exposure to iodinated contrast media. Notably, coronary CT is solely a diagnostic modality and in the event of a positive test an invasive coronary angiography and angioplasty would be needed, requiring a second exposure to iodinated contrast media. Another concern regarding most tests used for CV disease screening is the required radiation exposure in patients who will subsequently be at higher risk of cancer as a result of post-transplant immunosuppression.

## Post-transplantation diabetes mellitus

Previously, transplant immunosuppressive regimens depended on high doses of corticosteroids, which led to an increased risk of DM after transplant (up to 50% of transplant recipients ^41^). Immunosuppression has evolved to rely more on calcineurin inhibitors (CNIs) than on corticosteroids. Despite this, the incidence of PTDM did not decline as expected, leading to the discovery of the diabetogenicity of CNIs, particularly tacrolimus ^42^. However, since the early 2000s, the incidence of PTDM has declined ^16^, probably due to decreased rates of rejection episodes and reduced exposure to corticosteroids and CNIs ^42^. The 5-year incidence of PTDM according to the 2016 Scientific Registry of Transplant Recipients (SRTR) report is about 12% ^16^.

Both pre-transplant DM and PTDM are associated with higher risk of CV events, graft failure, and mortality ^6, 18, 43– 45^. Therefore, early diagnosis and treatment are necessary. However, post-transplant hyperglycemia is dynamic, demonstrated by the transient hyperglycemia seen early post-transplant and during rejection events, both situations requiring high doses of corticosteroids ^42, 46^. As a result, the consensus is to establish a diagnosis when the patient is stable on their maintenance immunosuppression regimen, with stable kidney function and no acute infections ^46^.

Risk factors for PTDM development include type of immunosuppression, ethnicity, obesity, hypomagnesemia, hepatitis C virus (HCV), and cytomegalovirus (CMV) infection ^18, 42, 47– 49^. Lifestyle modification, as well as adjusting the modifiable risk factors such as immunosuppression type and infections, should be taken into account before transplantation. For instance, successful HCV treatment has been associated with lower risk of PTDM ^50, 51^.

Obese patients obtain a clear benefit from kidney transplant compared with staying on dialysis ^52^, but high body mass index (BMI) is associated with a higher risk of PTDM and worse patient and allograft outcomes ^53– 55^. Hence, many transplant centers exclude patients with high BMI from listing (BMI cutoff varies from 35 to 45 kg/m ^2^ between transplant centers ^56^). Furthermore, a significant proportion of patients gain weight in the post-transplant period, likely because of the high doses of steroids and the liberalized diet ^57, 58^. Case series and small studies have reported promising results of bariatric surgery pre- and post-transplant ^59– 65^. Attention to the possibility of malabsorption induced by bariatric surgery (that is, *Roux-en-Y* gastric bypass) is important because of an increased risk of urolithiasis, oxalate deposition in the kidney, and the potential (but not yet proven by proper, large pharmacokinetic studies) decreased absorption of immunosuppressive medications ^61, 64, 66, 67^.

Changes in immunosuppression should be based on overall patient and allograft benefit rather than on the risk of PTDM development alone ^46^. Although tacrolimus has a higher risk of PTDM compared with cyclosporine A ^42, 68^, the former is generally preferred because of the lower risk of rejection and higher graft survival ^69^. The benefit of early corticosteroid withdrawal has been controversial; the largest randomized trial found no difference in PTDM development at 5 years post-transplant with corticosteroid maintenance versus early withdrawal ^70^. This contrasts with the findings of an earlier meta-analysis that showed a reduced risk of PTDM with early steroid withdrawal but also an increased risk of allograft rejection ^71^. When steroid withdrawal is chosen, the PTDM incidence is similar if steroids are given for 10 days versus an intraoperative bolus only, but the incidence of rejection is higher in the second group ^72^. Another potential strategy to decrease the risk of PTDM would be to use CNI-free regimens. Use of belatacept, a T-cell co-stimulation blocker, reduces the risk of PTDM by 39% compared with CNIs ^73^. Although mammalian target of rapamycin (mTOR) inhibitors are associated with a better glycemic profile than tacrolimus, they result in a worse lipid profile and higher rejection risk ^42^.

Treatment of DM in the post-transplant period includes lifestyle modification with particular attention to healthy weight maintenance as well as pharmacologic therapy. Owing to the lack of evidence derived from well-designed prospective clinical trials investigating differences in hard clinical end points such as mortality, allograft loss, and CV events in this population, the optimal pharmacologic agent in transplant recipients is not well established ^42^. In the early post-transplant period, it is recommended to treat hyperglycemia with insulin since it is the safest and most effective agent in the context of high corticosteroid doses ^46^. Furthermore, this approach appears to reduce the odds of developing PTDM by 73% in the first year post-transplant ^74^. After corticosteroid doses are reduced, treatment with oral anti-hyperglycemic agents is recommended, but the choice of specific agent should be individualized. Because of a lack of evidence, the most recent consensus recommendations were unable to propose a hierarchy of anti-hyperglycemic agents for PTDM ^46^. The most commonly used anti-hyperglycemic medications post-transplant include metformin, sulfonylureas (that is, glipizide and glimepiride), and meglitinides (that is, repaglinide). Newer medications such as DPP-4 inhibitors (that is, sitagliptin, linagliptin, and vildagliptin) and GLP-1 agonists (exenatide and liraglutide) have been proposed for PTDM given their ability to counteract the effects of CNIs and corticosteroids by increasing glucose-dependent insulin secretion and inhibiting glucagon secretion ^75^. In the general population, treatment with GLP-1 agonists decreases MACE ^76, 77^ but this has not yet been confirmed in the kidney transplant population. Although no long-term randomized trials of these agents using clinical end points have been carried out for PTDM, studies have demonstrated that they are safe and effective in controlling hyperglycemia in post-transplant patients ^78– 84^. Finally, SGLT-2 inhibitors (that is, empagliflozin, canagliflozin, and dapagliflozin) have been shown to be effective in the general population in controlling hyperglycemia, reducing proteinuria, slowing kidney function decline, promoting weight loss, decreasing blood pressure, and reducing CV risk ^85– 87^. To date, only case reports and case series have reported the use of SGLT-2 inhibitors in the post-transplant period, and the biggest series of 25 patients has only been reported in abstract form ^88– 90^. These small series have shown that the effect of SGLT-2 inhibitors post-transplant is similar to the general population in terms of glucose control, weight loss, and decrease in blood pressure ^88– 90^. However, no long-term data are available, and no benefits in hard outcomes such as death, MACE, or graft failure rate have been demonstrated in kidney transplant recipients. Furthermore, some features of these medications, including the increased risk of genitourinary infections, volume depletion, ketoacidosis, and amputations, are particularly concerning for kidney transplant recipients ^85, 86, 91, 92^. Given the retrospective nature of the studies carried out to date in the post-transplant period with SGLT-2 inhibitors and the small number of patients included, we cannot reach a definitive conclusion on their safety profile ^88– 90^. A prospective interventional trial is ongoing (EMPTRA-DM) and we anxiously await the results ^93^.

## Cancer

Solid organ transplant recipients have a twofold increased risk of cancer compared with the general population ^19^. The standardized incidence ratios (SIRs) for infection-related malignancies—that is, Epstein–Barr virus (EBV)-associated lymphoma, Kaposi sarcoma (KS), hepatocellular carcinoma, genital and gastric cancers—are significantly elevated in kidney transplant recipients ^19, 94^. However, dozens of other cancer types unrelated to infection are also more common in the transplant population; the highest SIR is for the following cancers: squamous cell cancers of the skin and lip, renal cell carcinoma (RCC), cholangiocarcinoma, and salivary gland cancer ^19^. Moreover, the incidence of lung and colorectal cancers, which are very common in the general population, is even higher in the transplant population ^19, 94^. In contrast, the SIR of prostate and breast cancer is not higher in transplant recipients compared with the general population ^19, 94^ ( Table 3). Therefore, screening and preventative measures should be implemented in all transplant candidates and recipients. However, evidence to support a specific screening strategy is often lacking. A recent systematic review of clinical practice guidelines (CPGs) on cancer screening in solid organ transplant recipients highlights this point by demonstrating significant discrepancies across 13 CPGs (including eight CPGs specific to kidney transplant). Explanations for these differences include authors’ interpretation of indirect data (that is, evidence of high incidence of a type of cancer but no evidence regarding the efficacy of the screening strategy) as well as lack of input from oncologists and public health and cancer screening experts ^95^. Further research is needed to formulate evidence-based cancer screening guidelines for this high-risk population.

**Table 3.** Incidence of cancer in solid organ transplant recipients as reported by Engels *et al.* ^19^.

Cancer site	Standardized incidence ratio (95% CI)
**Infection-related malignancies**	
Non-Hodgkin lymphoma	7.54 (7.17–7.93)
Liver	11.56 (10.83–12.33)
Stomach	1.67 (1.42–1.96)
Kaposi sarcoma	61.46 (50.95–73.49)
Oropharynx	2.01 (1.64–2.43)
Anus	5.84 (4.70–7.18)
Hodgkin lymphoma	3.58 (2.86–4.43)
Vulva	7.60 (5.77–9.83)
Cervix	1.03 (0.75–1.38)
Penis	4.13 (2.59–6.26)
Nasopharynx	0.96 (0.42–1.90)
Vagina	2.35 (0.94–4.84)
**Non-infection-related malignancies**	
Lung	1.97 (1.86–2.08)
Prostate	0.92 (0.87–0.98)
Kidney	4.65 (4.32–4.99)
Colorectum	1.24 (1.15–1.34)
Breast	0.85 (0.77–0.93)
Melanoma	2.38 (2.14–2.63)
Thyroid	2.95 (2.58–3.34)
Urinary bladder	1.52 (1.33–1.73)
Skin (non-melanoma, non- epithelial)	13.85 (11.92–16.00)
Pancreas	1.46 (1.24–1.71)
Lip	16.78 (14.02–19.92)
Plasma cell neoplasm	1.84 (1.52–2.20)
Acute myeloid leukemia	3.01 (2.45–3.65)
Larynx	1.59 (1.29–1.95)
Esophagus	1.56 (1.26–1.91)
Uterine corpus	0.86 (0.70–1.05)
Soft tissue, including heart	2.25 (1.74–2.87)
Salivary gland	4.55 (3.44–5.91)
Ovary	0.95 (0.72–1.24)
Small intestine	2.43 (1.80–3.20)
Brain	0.76 (0.55–1.01)
Testis	1.96 (1.40–2.67)
Intrahepatic bile duct	5.76 (4.08–7.91)
Chronic myeloid leukemia	3.47 (2.46–4.77)
Chronic lymphocytic leukemia	0.59 (0.38–0.89)
Gallbladder	2.00 (1.25–3.02)
Eye and orbit	2.78 (1.72–4.24)
Renal pelvis	2.05 (1.20–3.29)
Acute lymphocytic leukemia	2.06 (1.20–3.30)
Mesothelioma	1.30 (0.73–2.15)
Bones and joints	1.98 (1.09–3.33)
Other acute leukemia	2.20 (0.71–5.13)
Acute monocytic leukemia	2.35 (0.64–6.01)

CI, confidence interval.

One of the most important risk factors for cancer development is tobacco use, but until recently there was only limited evidence regarding the benefits of smoking cessation. Opelz and Döhler ^96^ found that the incidence of several cancers, most prominently lung cancer, significantly decreases with smoking cessation before transplant; however, incidences remain higher than in never-smokers. In the same study, smoking cessation before transplant also decreased the risk of graft loss and death ^96^. Hence, it is strongly recommended to encourage smoking cessation at the time of kidney transplant candidacy evaluation.

Immunosuppression is a prominent risk factor for cancer development, but whether specific immunosuppressive agents confer higher risk than others is an area of active study. Azathioprine increases the risk of squamous cell carcinoma but not other types of skin cancer ^97, 98^, whereas mycophenolate may be protective ^98^. Conflicting results have been obtained in studies comparing tacrolimus with cyclosporine in regard to the risk of malignancy ^99– 104^. Belatacept confers increased risk of post-transplant lymphoproliferative disorder, particularly in EBV-seronegative patients ^105– 107^. In regard to induction therapy, alemtuzumab increases the risk of non-Hodgkin lymphoma, other virus-related cancers, and colorectal and thyroid cancer ^99^ whereas anti-lymphocyte globulin increases the risk of melanoma but surprisingly does not alter the risk of lymphomas ^99, 108^. Basiliximab did not increase the risk of cancers evaluated in this study ^99^.

mTOR inhibitors (that is, sirolimus and everolimus) suppress growth and proliferation in malignant cells ^109– 111^. Hence, mTOR inhibitor–based regimens have been used *de novo* to prevent cancer development in transplant patients with high cancer risk and conversion to an mTOR inhibitor–based regimen is often considered if cancer is diagnosed. Whereas a 2014 meta-analysis showed a decreased risk of malignancy by 40% (driven mainly by the decreased risk of non-melanoma skin cancer) ^112^, recent large studies have failed to show a difference in overall malignancy risk between patients on mTOR inhibitor–based immunosuppression and other regimens ^113– 116^. Some studies have not included non-melanoma skin cancer in their analysis, but most of the studies that did have found a decreased risk of non-melanoma skin cancer (particularly basal cell carcinoma) ^112, 114, 116– 119^. Nonetheless, mTOR inhibitor–based immunosuppression regimens have been associated with increased risk of mortality ^112, 115^ and post-transplant lymphoproliferative disorder ^120, 121^. Therefore, it is not recommended to change to mTOR inhibitor–based regimens after a cancer diagnosis. The only situation in which conversion to mTOR inhibitor–based regimen is recommended is in transplant patients diagnosed with KS ^122^ because of reports of complete regression of the KS lesion after conversion ^123– 125^.

Cancer immunotherapy is an emerging field requiring particular attention in kidney transplant recipients. Checkpoint inhibitors, which target the programmed cell death pathway (PD-1 and PD-L1) and the cytotoxic T-lymphocyte-associated antigen-4 (CTLA-4), are effective in the treatment of several types of cancers, including melanoma, RCC, and non-small cell lung cancer ^126, 127^. However, case reports and series have reported a high risk of allograft rejection and loss with monoclonal antibodies against PD-1 but the incidence of this outcome is unknown ^127– 129^. Conversely, this outcome is seen less frequently with agents targeting CTLA-4 ^127^. A recent review found 17 reported solid organ transplant recipients (11 kidney, three liver, and three heart recipients) treated with these agents. One (16%) of six patients treated with the CTLA-4 inhibitor ipilimumab had allograft rejection, compared with 5 (62%) of 8 patients treated with PD-1 inhibitors, and 2 (66%) of 3 patients treated with CTLA-4 followed by PD-1 inhibitors ^130^. A similar pattern was seen in the subset of kidney transplant recipients alone ^130^. Further research is necessary to clarify the safety and effectiveness of checkpoint inhibitors in transplant recipients and for development of regimens that minimize the risk of allograft rejection, such as combining these agents with mTOR, BRAF, mitogen-activated protein kinase (MEK), and Bruton’s tyrosine kinase (BTK) inhibitors ^130^.

## Infectious complications

Infectious complications are common during the post-transplant period and account for 13% of overall mortality in kidney transplant recipients ^17^. The degree of immunosuppression and epidemiological exposures are the main determinants of the risk of infections. Transplant infectious disease experts typically divide the post-transplant period into three roughly different intervals ^131^:

(1) first month post-transplant, when infections are either a complication of the surgery/hospitalization or pre-existing in the donor or recipient;
(2) one to six months post-transplant; this is the period when immunosuppression is often the highest and opportunistic infections ( *Pneumocystis jirovecii* pneumonia, CMV or other herpes virus infections, mycobacterial infections, and so on) often happen, warranting prophylactic measures to prevent such infections;
(3) after 6 to 12 months post-transplant, when immunosuppression is usually more stable and lower than in previous periods.

This timeline is obviously altered by heightened immunosuppression due to rejection episodes. During all of these periods, kidney transplant recipients are also at higher risk of “garden-variety” infections such as community-acquired pneumonia, urinary tract infections, and so on.

In this article, we decided to focus on three particularly important subjects that either have changed dramatically in the last few years (that is, HCV) or warrant special attention in kidney transplant recipients (that is, CMV and BK virus), despite not being the most common infectious complications during the post-transplant period.

### Hepatitis C virus

HCV infection is associated with higher incidences of CKD, faster progression to ESRD, and higher morbidity and mortality ^132– 134^. The prevalence of HCV in the ESRD population is significantly higher than in the general population, a finding which continues after transplantation ^135^. HCV infection is associated with worse allograft outcomes (that is, allograft rejection, chronic allograft nephropathy and decreased graft survival), hepatic complications (that is, cirrhosis and hepatocellular carcinoma), PTDM, CV disease, *de novo* or recurrent glomerulonephritis, and overall worse patient survival ^136– 141^. Nevertheless, HCV-infected patients who receive a transplant have significantly lower morbidity and mortality than HCV-infected patients who remain on the waiting list ^142– 144^.

Historically, treatment of HCV infection included interferon or pegylated interferon with or without ribavirin. Owing to a high rate of allograft rejection and loss, interferon is not recommended in transplant recipients ^145^, and ribavirin causes hemolytic anemia in patients with low GFR ^146^. The recent development of direct-acting antivirals (DAAs) has dramatically changed HCV treatment, making interferon and ribavirin treatment essentially obsolete. Several all-oral, interferon-free DAA regimens are highly effective and safe in CKD stages 4–5, ESRD, and kidney transplant populations with sustained virological response (SVR) rates of 90% to 100% ^147– 163^. DAA treatment in kidney transplant recipients results in SVR rates ranging from 98% to 100% ^153– 163^. However, 36% of transplant patients require CNI dose adjustment, but no increased risk of acute rejection has been reported ^153– 163^. Screening for hepatitis B virus (HBV) infection is recommended before starting HCV treatment given the risk for HBV reactivation with DAAs ^164^.

The availability of HCV-positive deceased donor kidneys has increased dramatically as a result of the opioid epidemic in the USA. Moreover, the waiting time for an HCV-positive kidney is much lower than for an HCV-negative kidney as these organs are currently not offered to HCV-negative transplant candidates ^157, 158, 165– 167^. This leads to two important questions: (1) Should HCV-positive candidates be treated with DAAs before or after kidney transplantation? (2) Is it safe to transplant HCV-positive organs into HCV-negative recipients?

In terms of timing of HCV infection treatment, the most important factors to consider are DAA accessibility, the availability of HCV-positive organs, and the waiting time reduction in the area where the patient would be transplanted. If using HCV-infected donor kidneys results in a significant reduction in waiting time and DAAs are available, most patients would benefit from transplantation first followed by HCV treatment after transplantation. However, situations exist where treatment of HCV infection is indicated before transplant, including severe extrahepatic HCV manifestations such as mixed cryoglobulinemia syndrome, compensated cirrhosis with high risk of liver disease progression, and a living donor available only after 24 weeks (where there is no benefit of delaying the 12-week treatment and 12-week post-treatment monitoring for SVR) ^135^.

Two trials evaluating the safety of transplanting HCV-positive organs to HCV-negative recipients have been published recently. In the THINKER (Transplanting Hepatitis C kidneys Into Negative Kidney Recipients) trial ^166, 168^, 20 HCV-negative patients have been transplanted with HCV-infected kidneys followed by initiation of elbasvir/grazoprevir on post-transplant day 3. All recipients developed a positive HCV viral load post-transplant, but within 4 weeks of treatment the virus was undetectable, and 100% of patients achieved SVR at 12 weeks ^166, 168^. At 1 year of follow-up, allograft function, blood pressure, and proteinuria were excellent ^155^. Another 10 HCV-negative recipients have been transplanted with HCV-positive kidneys in the EXPANDER-1 (Exploring Renal Transplants Using Hepatitis C Infected Donors for HCV-Negative Recipients) trial in which elbasvir/grazoprevir was started immediately before transplantation. Only three patients had detectable HCV RNA early post-transplant, and 100% had negative HCV viral load at 12 weeks ^169, 170^. Both trials showed a good safety profile of elbasvir/grazoprevir early post-transplant and good early allograft outcomes ^166, 168– 170^. Currently, this practice is recommended in the controlled setting of clinical trials only, but if the results of the THINKER and EXPANDER-1 trials are confirmed in larger studies, it might become a widespread practice that would expand the donor pool.

### Cytomegalovirus

CMV remains a frequent infectious complication after kidney transplantation. Active CMV infection can lead to a viral syndrome (CMV syndrome) or tissue-invasive disease—including colitis, pneumonitis, nephritis, hepatitis, encephalitis, and retinitis—or both. Furthermore, CMV infection has “indirect effects”, including increased risk of allograft failure ^131, 171^.

The most important risk factor is CMV serostatus in the donor and recipient, and the highest risk is in the CMV IgG-negative recipient transplanted with a CMV IgG-positive organ ^172^. Another risk factor is level and type of immunosuppression; incidence is higher in patients induced with lymphocyte-depleting agents (that is, thymoglobulin), and incidence is lower with mTOR inhibitor–based chronic immunosuppression regimens ^173– 179^.

CMV prevention strategies are commonly used post-transplant. The main approaches are universal prophylaxis or pre-emptive therapy. Universal prophylaxis entails administration of antiviral drugs (most commonly valganciclovir) to all patients or those at higher risk of CMV infection starting the first 10 days post-transplant and continuing for 3 to 6 months. Treatment duration is guided by the specific CMV status of donor and recipient. This approach is easier to implement and prevents early CMV infection; however, late CMV infection is more common with this approach, drug costs may be high, and patients often experience drug-induced adverse events, including leukopenia and hepatitis, among others ^172^. Pre-emptive therapy involves CMV viral load monitoring at regular intervals (most often weekly), and therapy is started only when a specific viral load threshold is crossed. Because of the variability of the tests, no universal threshold to initiate therapy has been defined ^180, 181^. This approach eliminates the possibility of drug side effects and prevents late CMV infection better, but early CMV infection is more frequent. Moreover, this approach may be more difficult to implement and the costs of frequent monitoring might also be high ^172^. A randomized trial comparing both approaches found no difference in the incidence of CMV disease ^182^. The role of screening for CMV viremia later after transplantation is not well established.

Treatment of CMV syndrome or tissue-invasive disease consists of oral valganciclovir or intravenous ganciclovir. In the Study of Valcyte Compared to Ganciclovir in Patients with Cytomegalovirus Disease who are Solid Organ Transplant Recipients (VICTOR trial) ^183^, 321 solid organ transplant patients (>70% kidney transplant recipients) with CMV viremia, CMV syndrome, or CMV disease were randomly assigned to ganciclovir or valganciclovir. Viremia eradication and treatment success were similar in the two groups. Nevertheless, international consensus guidelines still recommend intravenous ganciclovir as the initial treatment for patients with life-threatening CMV infection ^172^. Reduction of the intensity of immunosuppression is also associated with higher CMV eradication rates ^184^.

Drug resistance needs to be suspected when there is persistent CMV viremia or disease despite prolonged antiviral therapy (6 or more weeks of cumulative drug exposure or more than 2 weeks of ongoing full-dose therapy) ^172^. Genotypic assays to detect mutations are used to test for resistance. In 90% of patients, mutation of the UL97 kinase gene appears first with varying degrees of resistance to ganciclovir but does not confer cidofovir or foscarnet resistance. UL54 mutations evolve later, conferring increased ganciclovir resistance and possibly cidofovir or foscarnet resistance or both. Depending on the mutation encountered, treatment with high doses of ganciclovir, foscarnet, or cidofovir could be indicated, but no controlled trials have defined the best intervention in these cases ^172^.

### BK virus

BK virus is a polyomavirus that is highly seroprevalent in humans but causes disease only in immunocompromised patients. Following kidney transplantation, BK virus causes tubulointerstitial nephritis in 1% to 10% of patients ^185– 188^. The most important risk factor for BK virus nephropathy development is the level of immunosuppression, but recent studies have demonstrated the importance of BK virus–specific T-cell functionality ^189, 190^ and BK virus seroprevalence in donors and recipients, and risk is higher in those donor-positive/recipient-negative pairs ^191, 192^. Antibody-depleting induction therapy increases the risk of BK virus nephropathy, whereas mTOR inhibitor reduces the risk ^193^.

Screening of BK virus nephritis relies on testing for viral replication in urine and blood. Viral replication in urine is tested with urine cytology looking for decoy cells or by polymerase chain reaction (PCR), whereas plasma or whole blood viral replication is confirmed by PCR. In general, the correlation with BK virus–associated nephropathy is higher for viremia (positive BK PCR in blood), lower for viruria (positive BK PCR in urine), and lowest for urine cytology ( Table 4) ^185, 194– 198^. The KDIGO guidelines on kidney transplant recipients recommend urine or blood PCR monthly for the first 3 to 6 months post-transplant and then every 3 months until the end of the first post-transplant year ^122^. However, other international consensus guidelines suggest continuing screening every 3 months until the end of the second year post-transplant and yearly thereafter ^186, 199^. Definitive diagnosis is made with histology on kidney allograft biopsy showing tubulointerstitial nephritis with cytopathic changes and positive immunohistochemistry for SV40 ^131^.

**Table 4.** Non-invasive diagnostic tests for BK virus–associated nephropathy.

Test	Threshold value	Sensitivity	Specificity	PPV	NPV
Decoy cells ^185, 194– 197^	>10 cells/cytospin	25–100%	71–96%	5–57%	97–100%
Urine BK PCR ^197, 198^	>1×10 ^7^ copies/mL	100%	92–96%	31–67%	100%
Blood/plasma BK PCR ^185, 195, 197, 198^	>1×10 ^4^ copies/mL	100%	88–96%	50–82%	100%

NPV, negative predictive value; PCR, polymerase chain reaction; PPV, positive predictive value.

The cornerstone of therapy is reduction of immunosuppression ^121^. However, the specific strategy of immunosuppression reduction is not well stablished and is mainly center-specific. A common practice is withdrawal of the antimetabolite drug (usually mycophenolate) and decrease of CNI dosing by 50%. Alternative approaches have been to stop tacrolimus and initiate either cyclosporine or an mTOR inhibitor. However, the evidence to support any of these approaches is low ^200– 204^. Some agents with antiviral properties have been suggested. Adding cidofovir or leflunomide does not increase graft survival ^205^. Treatment with intravenous immunoglobulin (IVIG) is promising based on the fact that many formulations of IVIG have neutralizing BK antibodies ^206^ and several case series have described its efficacy ^207, 208^, but large randomized controlled trials are needed before its widespread use can be recommended. Although quinolones have been reported to have anti-BK properties, randomized trials have shown no benefit of adding levofloxacin ^209, 210^.

## Conclusions

Kidney transplant recipients have a high risk of complications due to adverse events of potent immunosuppressive medications and their pre- and post-transplant complex medical history. It is important for the clinician taking care of these patients to be aware of the most common complications encountered in the post-transplant period and how to screen, diagnose, and treat them. A multidisciplinary team approach is often required given the multiple complications that fall into different medical and surgical specialties in kidney transplant recipients.
